# Comparison of the prevalence of osteoporosis in people with spinal cord injury according to bone mineral density reference values for the diagnosis of osteoporosis: a retrospective, cross-sectional study

**DOI:** 10.1186/s12891-024-07184-9

**Published:** 2024-01-26

**Authors:** Jisun Lim, Onyoo Kim

**Affiliations:** 1 Department of Clinical Research on Rehabilitation, National Rehabilitation Center, 58, Samgaksan-ro, Gangbuk-gu, Seoul, 01022 Republic of Korea; 2 Department of Rehabilitation Medicine, National Rehabilitation Center, 58, Samgaksan-ro, Gangbuk-gu, Seoul, 01022 Republic of Korea

**Keywords:** Spinal cord injury, Bone mineral density, T-score, Osteoporosis, Prevalence

## Abstract

**Background:**

Spinal cord injury (SCI) is a complex cause of rapid low bone mass that easily predisposes the affected individuals to osteoporosis-induced fractures. Several studies have investigated osteoporosis pathophysiology in SCI; however, those associated with its diagnosis in SCI are limited. Additionally, errors in osteoporosis diagnosis and its prevalence vary based on the bone mineral density (BMD) reference values (BMDRV), and no studies have reported BMDRV application for osteoporosis diagnosis in individuals with SCI. Therefore, this study aimed to compare the prevalence of osteoporosis among Korean adults aged ≥ 50 years with SCI according to BMDRV for diagnosing osteoporosis.

**Methods:**

Overall, 855 patients with SCI who underwent BMD tests of the lumbar spine, femoral neck, and total hip at the National Rehabilitation Center (NRC) in Korea between 2010 and 2020 were included in this retrospective cross-sectional study. Osteoporosis was diagnosed in patients with SCI by comparing the differences in prevalence, diagnostic consistency, and risk factors according to the region-based BMDRV of the dual-energy x-ray absorptiometry (DXA) manufacturer and international BMDRV based on the Third National Health and Nutrition Examination Survey (NHANES III) data of females aged 20–29 years.

**Results:**

The prevalence of osteoporosis according to the T-score provided by a single reference population of the NHANES III (TNHA) (PONHA) (males: 26.69%; females: 69.35%) was significantly higher in females and males than that according to the T-scores provided by the DXA manufacturer (TDXA) (PODXA) (males: 15.32%; females: 43.15%). The lumbar spine and femoral neck were major osteoporosis diagnosis sites for the PODXA and PONHA, respectively. Risk factors for osteoporosis differed based on the probability of osteoporosis (also known as the OZ ratio) according to the BMD criteria; however, the risk factors were similar according to old age, female sex, low body mass index (BMI), and long SCI period. No significant relationship was noted between the different SCI-related clinical factors (*p* > 0.05).

**Conclusions:**

The osteoporosis diagnostic site and prevalence in SCI differed according to the regional-based TDXA and international standards of the TNHA. Therefore, further studies on BMDRV are warranted to establish accurate diagnostic criteria for osteoporosis prevention in patients with SCI.

## Background

Spinal cord injury (SCI) is a complex cause of rapid low bone mass, and individuals with SCI are more easily predisposed to osteoporosis-induced fractures than those without disabilities. Therefore, accurate early diagnosis and treatment are important for osteoporosis prevention [1–3].

The World Health Organization (WHO) recommends that osteoporosis should be diagnosed by measuring bone mineral density (BMD) using central dual-energy X-ray absorptiometry (DXA) among postmenopausal females and males aged ≥ 50 years and that T-scores should be calculated based on the mean (average) and standard deviation (SD) of BMD in the baseline group; when the T-score is ≤˗2.5, the condition is diagnosed as osteoporosis [4, 5].

Major global osteoporosis guidelines (World Health Organization Scientific Group, 2007; International Society of Clinical Densitometry 2019; and National Osteoporosis Guideline Group, 2022) recommend using the BMD data of Caucasian females aged 20–29 years from the National Health and Nutrition Survey (NHANES) as international BMD reference values (BMDRV) since the data derived from such large-scale sample size is highly reliable and the BMD of this population group is high, indicating that their data reflect suitable and stable reference values [4, 6–9].

However, many studies have reported that the BMDRV differ according to age, sex, and race; therefore, regional-based BMDRV have been applied [5, 10–12]. For instance, Asians generally have a lower average BMD than Caucasians; therefore, osteoporosis can be overdiagnosed using the NHANES as BMDRV, depending on the skeletal site [13, 14].

Major DXA manufacturers set the BMDRV based on sex and region. Hologic and Lunar Prodigy Advance DXA equipment are used in Korean clinical trials and for diagnosing osteoporosis by deriving T-scores using the BMDRV of Japanese and some Koreans [15–17].

However, patients with SCI are a high-risk group with a higher prevalence of osteoporosis than those without disabilities [1], and their major osteoporotic skeletal sites differ [18–22] from those of individuals without disabilities. Charmetant et al. [23] reported that many studies have been conducted on osteoporosis pathophysiology in individuals with SCI, while those related to its diagnosis are limited [23]. Additionally, errors in osteoporosis diagnosis and prevalence differ according to the BMDRV [24]; however, to the best of our knowledge, no studies have reported on the use of BMDRV for diagnosing osteoporosis in individuals with SCI.

Therefore, this study aimed to establish BMDRV for diagnosing osteoporosis in individuals with SCI by comparing the differences in the prevalence, diagnostic consistency, and risk factors of osteoporosis according to the region-based BMDRV of the DXA manufacturer and the international BMDRV using the Third NHANES data of females aged 20–29 years.

## Methods

### Study design and participants

**Fig. 1:**
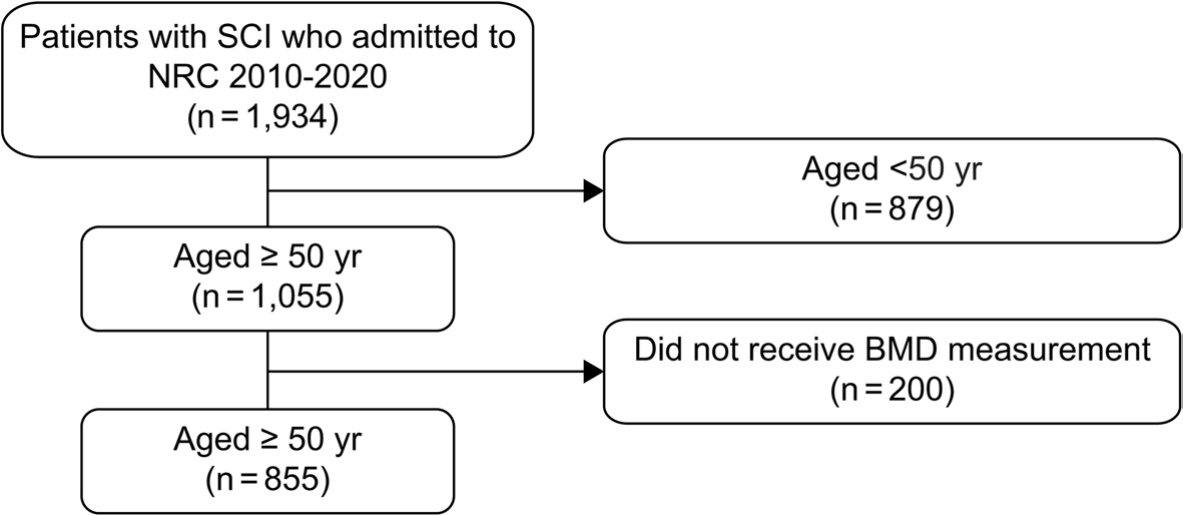
Flow diagram for the identification of the study population. NRC, National Rehabilitation Center; BMD, bone mineral density

We conducted a retrospective, cross-sectional study targeting patients with SCI aged ≥ 50 years who were admitted to the National Rehabilitation Center (NRC) in Korea, where BMD tests were conducted between 2010 and 2020. During this period, 1,934 patients were hospitalized for SCI. Among them, 855 patients aged ≥ 50 years underwent DXA. We obtained their data on age, sex, body mass index (BMI), health behavior (smoking history and alcohol consumption habits), time from injury, etiology of injury, main diagnosis, neurologic level of injury (NLI), American Spinal Injury Association impairment scale (AIS) score [14], and BMD from the electronic medical records and the picture archiving and communication system (Fig. 1). The Institutional Review Board of the NRC provided ethical approval (NRC-2021-01-011) and waived the requirement for informed consent due to the study’s retrospective nature.

### Diagnosis of osteoporosis

The DXA devices used to diagnose osteoporosis at the NRC were the Lunar Prodigy Advance® (GE Healthcare, USA) between January 1, 2010, and January 20, 2019, and the Hologic Discovery® (Hologic, USA) between January 21, 2019, and December 31, 2020. Consequently, the target BMD was determined by calculating the systemic differences in BMD values according to the manufacturer of the densitometers. The target BMD value measured using the Lunar Prodigy Advance® was also collectively converted to the Hologic Discovery® standards as follows: lumbar spine, Hologic Inc. BMD = 0.918 × GE Lunar BMD − 0.038; femoral neck, Hologic Inc. BMD = 0.8638 × GE Lunar BMD − 0.039; and total hip, Hologic Inc. BMD = 0.971 × GE Lunar BMD − 0.037 [25].

Osteoporosis was diagnosed using a standardized method suggested by the WHO and defined as normal (T≥-1), osteopenia (-1 < T < 2.5), and osteoporosis (T≤-2.5) for each measurement area according to the T-score of postmenopausal females and males aged ≥ 50 years. The T-score of any of the measurement areas (lumbar spine, femoral neck, and total hip) was ≤˗2.5 [21].

Furthermore, the T-score derivations were categorized into T-scores provided by the DXA manufacturer (TDXA) obtained from the region-based BMDRV of the DXA manufacturer. The BMDRV used by the manufacturer were derived from the reference values of Koreans and Japanese for the Lunar and Hologic devices, respectively [15–17]. Additionally, the T-scores provided by the Third NHANES (TNHA) reference population were derived from the international BMDRV using the Third NHANES data of females aged 20–29 years. The TNHA was calculated as follows: lumbar spine, lumbar_Isbmd_Hologic-1.047)/0.110; femoral neck, femoral neck_Isbmd_Hologic-0.86)/0.12; and total hip, total hip_Isbmd_Hologic-0.94)/0.122 [26, 27].

### Statistical analysis

Descriptive statistics were used to confirm the demographic and SCI-related disability characteristics of participants. Continuous and categorical variables are presented as mean and SD and numerical value (N) and percentage (%), respectively. The average T-score, osteoporosis prevalence, and risk factors were compared based on sex to compare the results of osteoporosis diagnosis according to the two BMDRV. Moreover, to determine if a significant difference exists in the mean T-score of the two BMDRV, a corresponding sample t-test (Wilcoxon signed-rank test) was performed after normality verification. Furthermore, a McNemar test was performed to determine the difference in osteoporosis prevalence, the category agreement was confirmed using Cohen’s Kappa statistic, and the osteoporosis prevalence was confirmed using the chi-square or Fisher’s exact test.

The difference in osteoporosis risk factors was confirmed using biologically reasonable variables (age, sex, BMI, health behavior, and SCI-related clinical factors). For each variable, multiple logistic regression analysis was performed after confirming the degree of significance through correlation analysis (*p* < 0.05). All statistical analyses were performed using the SAS software version 9.4 (SAS Institute Inc., Cary, NC, USA), and statistical significance was set at *p* < 0.05.

## Results

### Demographic and lifestyle characteristics

Table 1 presents the characteristics of the study participants. Of the 855 individuals analyzed, 70.99% (*n* = 607) and 29.01% (*n* = 248) were males and females, respectively, with an average age of 62.35 ± 8.67 years. Moreover, the average BMI was 22.96 ± 3.05 kg/m^2^, and 71.93% (*n* = 615) of participants had a normal BMI.

**Table 1:** Demographics and baseline clinical characteristics of participants

Characteristics	*N* = 855	
	Mean (SD)	*N* (%)
Age (years)	62.35 (8.67)	
50–59		383 (44.80)
60–69		290 (33.92)
70 +		182 (21.29)
Sex		
Male		607 (70.99)
Female		248 (29.01)
BMI (kg/m^2^)	22.96 (3.05)	
Underweight (< 18.5)		44 (5.15)
Normal (18.5–24.9)		615 (71.93)
Overweight (25.0–29.9)		181 (21.17)
Obese (≥ 30.0)		15 (1.75)
Smoking history		
Current smoking		135 (15.79)
Current non-smoking		96 (11.23)
Non-smoking		620 (72.51)
Unknown		4 (0.47)
Alcohol drinking		
Binge		6 (0.70)
Social		136 (15.91)
None		713 (83.39)
Time from injury (months)		
< 12 months		660 (77.19)
≤ 12 months		195 (22.81)
Etiology of injury		
Traumatic		532 (62.22)
Non-traumatic		294 (34.39)
Unknown		29 (3.39)
Main diagnosis		
Tetraplegia		508 (59.42)
Paraplegia		347 (40.58)
NLI		
Cervical		501 (58.60)
Thoracic		249 (29.12)
Lumbar		104 (12.16)
Sacrum		1 (0.12)
AIS		
A		176 (20.58)
B		97 (11.35)
C		180 (21.05)
D		385 (45.03)
E		2 (0.23)
Unknown		15 (1.75)

*Abbreviations*: *AIS* American Spinal Injury Association impairment scale, *BMI* Body mass index, *N* Number, *NLI* Neurologic level of injury, *SD* Standard deviation

Furthermore, participants had relatively good health behavior, without a smoking history (72.51%; *n* = 620) or alcohol consumption (83.39%; *n* = 713). In total, 77.19% (*n* = 660) of participants had SCI within 12 months. Participants with tetraplegia and paraplegia were 59.42% (*n* = 508) and 40.58% (*n* = 347), respectively. Lastly, the AIS classifications were A (*n* = 176; 20.58%), B (*n* = 97; 11.35%), C (*n* = 180; 21.05%), D (*n* = 385; 45.03%), and E (*n* = 2; 0.23%).

### Average T-score and prevalence of osteoporosis based on the diagnosis area and BMDRV

**Fig. 2:**
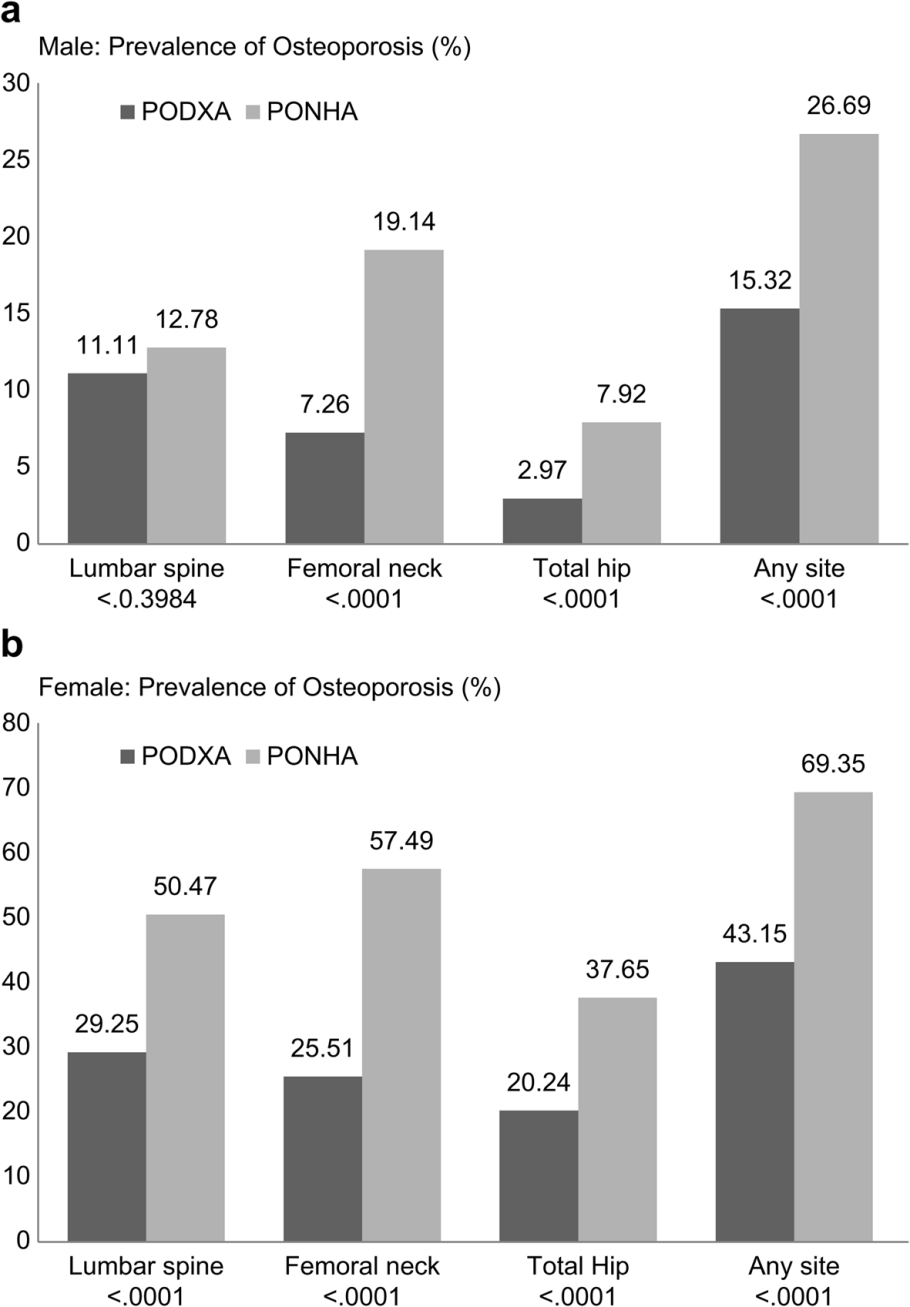
Osteoporosis prevalence according to two references based on the skeletal site. **a** Male: prevalence of osteoporosis (%). **b** Female: prevalence of osteoporosis (%)

Table 2 presents the average BMD area of the lumbar spine, femoral neck, and total hip for males and females, which were collectively adjusted for the Hologic device and the T-score averages according to the two BMDRV. Figure 2 shows the prevalence of osteoporosis according to the diagnosis site. The average BMD area differed according to the measurement site, and the average BMD area of the femoral neck was low in both males and females.

**Table 2:** BMD and T-score average according to two different BMDRV

Category		BMD Mean (SD)	TDXA Mean (SD)	TNHA Mean (SD)	*p* ^b^
Male	Lumbar spine (*N* = 540)	0.945 (0.165)	-0.913 (1.454)	-0.932 (1.496)	0.4862
	Femoral neck (*N* = 606)	0.678 (0.143)	-1.000 (1.186)	-1.511 (1.191)	< 0.0001***
	Total hip (*N* = 606)	0.851 (0.157)	-0.272 (1.201)	-0.732 (1.290)	< 0.0001***
	Any site^a^ (*N* = 607)		-1.430 (1.067)	-1.785 (1.102)	< 0.0001***
Female	Lumbar spine (*N* = 212)	0.794 (0.139)	-1.738 (1.225)	-2.297 (1.259)	< 0.0001***
	Femoral neck (*N* = 247)	0.564 (0.123)	-1.762 (1.143)	-2.466 (1.029)	< 0.0001***
	Total hip (*N* = 247)	0.692 (0.146)	-1.515 (1.267)	-2.028 (1.202)	< 0.0001***
	Any site^a^ (*N* = 248)		-2.202 (1.117)	-2.808 (1.063)	< 0.0001***

*Abbreviations*: *BMD* Bone mineral density, *BMDRV* Bone mineral density reference value, *DXA* Dual-energy X-ray absorptiometry, *NHANES* National Health and Nutrition Examination Survey, *N* Number, *SD* Standard deviation, *TDXA* T-score provided by the DXA manufacturer, *TNHA* T-score provided by a single reference population of the NHANES

^a^The minimum T-score of the lumbar spine, femoral neck, and total hip

^b^Wilcoxon’s signed-rank test

For *, **, and ***, the mean difference is significant at the 0.05, 0.01, and 0.001 levels, respectively

Statistical significance is set at *p* < 0.05

When comparing the T-score average for each measurement area according to the BMDRV, males had a lower average TNHA than average TDXA and a significant difference in the femoral neck and total hip (*p* < 0.0001), but not in the lumbar spine (*p* < 0.4862). Osteoporosis prevalence was also significantly higher in the prevalence of osteoporosis according to the TNHA (PONHA) than in the prevalence of osteoporosis based on the TDXA (PODXA) in all osteoporosis prevalence (*p* < 0.0001) except in the lumbar spine (*p* < 0.3984).

In females, the average TNHA at all sites was significantly lower than the average TDXA at all sites (*p* < 0.0001). Moreover, osteoporosis prevalence was similar, and PONHA was significantly higher in all sites than PODXA (*p* < 0.0001).

**Fig. 3:**
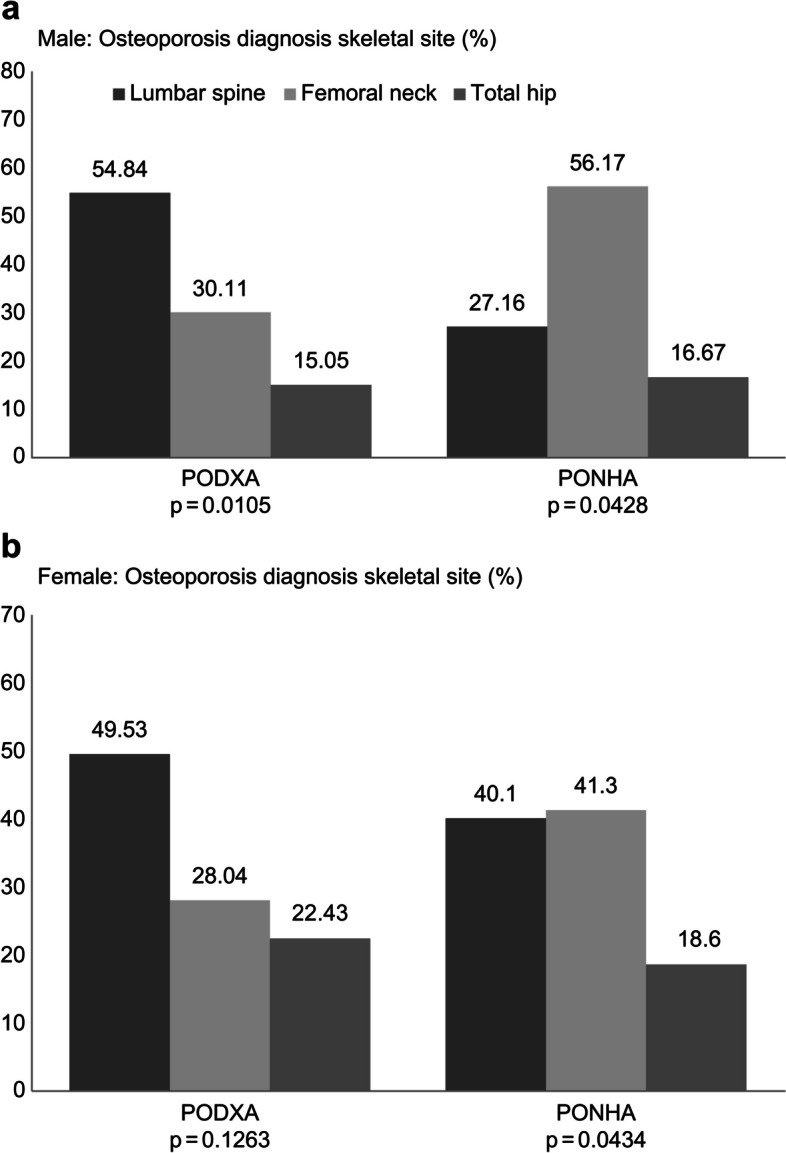
Skeletal site of the osteoporosis diagnosis according to two different references based on any site. **a** Male: skeletal site for the osteoporosis diagnosis (%). **b** Female: skeletal site for the osteoporosis diagnosis (%)

Figure 3 shows the schematic of the diagnostic site and any site according to the two BMDRV. According to the PODXA at any site, the diagnosis areas were the lumbar spine (males: 54.84%; females: 48.59%), femoral neck (males: 30.11%; females: 28.04%), and total hip (males: 15.05%; females: 22.43%). However, according to the PONHA, the diagnosis areas were the femoral neck (males: 56.17%; females: 41.3%), lumbar spine (males: 27.16%; females: 40.1%), and total hip (males: 16.67%; females: 18.6%).

### Prevalence of osteoporosis categorized by age according to BMDRV and consistency of the osteoporosis diagnosis using the two BMDRV

Table 3 presents the diagnostic consistency of the three categories of osteoporosis determination according to the two BMDRV criteria. Matching occurred for 76.94% and 64.92% of males and females, respectively, and Cohen’s Kappa were 0.6815 (substantial) and 0.5158 (moderate) for males and females, respectively. However, according to the McNemar test, the null hypothesis, suggesting that the significance level was *p* < 0.0001 and that the prevalence was similar for males and females, could be rejected.

**Table 3:** Agreement in the identification and establishment of osteoporosis using TDXA and TNHA cut-off values

Based on the TNHA	Based on the TDXA			Total *N* (%)		
	Normal	Osteopenia	Osteoporosis		*p*^*a*^	*k*^*b*^
	*N* (%)	*N* (%)	*N* (%)			
Male					< 0.0001***	0.6815
Normal	124 (20.43)	1 (0.16)	0 (0.00)	125 (20.59)		
Osteopenia	66 (10.87)	252 (41.52)	2 (0.33)	320 (52.72)		
Osteoporosis	1 (0.16)	70 (11.53)	91 (14.99)	162 (26.69)		
Total	191 (31.47)	323 (53.21)	93 (15.32)	607 (100)		
Female					< 0.0001***	0.5158
Normal	16 (6.45)	0 (0.00)	0 (0.00)	16 (6.45)		
Osteopenia	22 (8.87)	38 (15.32)	0 (0.00)	60 (24.19)		
Osteoporosis	0 (0.00)	65 (26.21)	107 (43.15)	172 (69.35)		
Total	38 (15.32)	103 (41.53)	107 (43.15)	248 (100)		

*Abbreviations*: *TDXA* T-score provided by the DXA manufacturer, *TNHA* T-score provided by a single reference population of the NHANES

^a^McNemar test

^b^Kappa

For *, **, and ***, the mean difference is significant at the 0.05, 0.01, and 0.001 levels, respectively. Statistical significance is set at *p* < 0.05

**Fig. 4:**
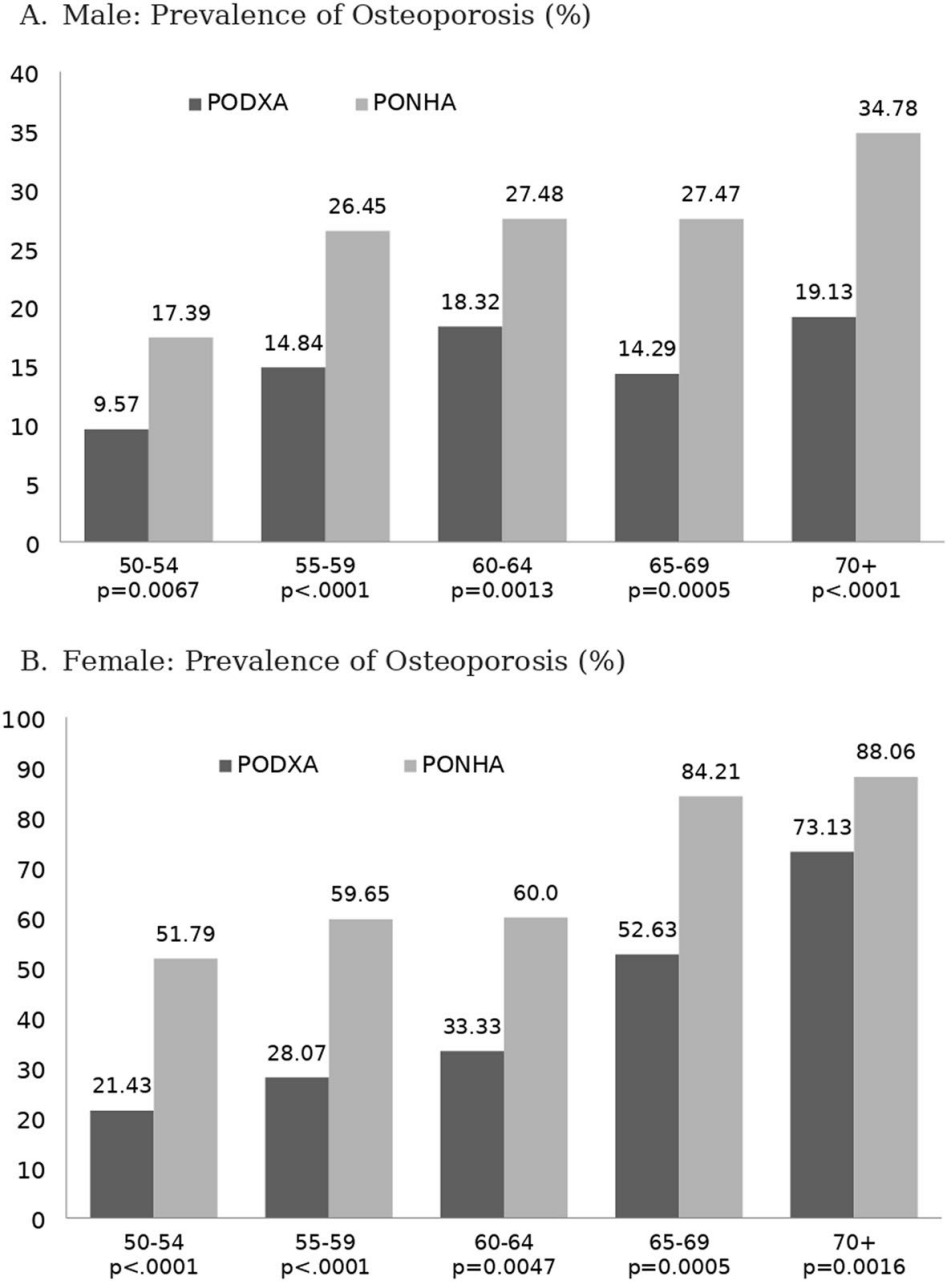
Prevalence of osteoporosis according to two different references based on age. **a** Male: prevalence of osteoporosis (%). **b** Female: prevalence of osteoporosis (%)

Table 4 presents the prevalence of osteoporosis, which is classified into three categories (normal, osteopenia, and osteoporosis) based on the two BMDRV for males and females categorized by age (Fig. 4). Additionally, in all age groups of males and females, the PONHA showed a higher prevalence of osteoporosis than the PODXA, with a significant difference (*p* < 0.05).

**Table 4:** Prevalence of osteopenia and osteoporosis according to two different BMDRV based on age groups

Sex	Age	Normal		Osteopenia		Osteoporosis		*p* ^a^
	(years)	PODXA *N* (%)	PONHA *N* (%)	PODXA *N* (%)	PONHA *N* (%)	PODXA *N* (%)	PONHA *N* (%)	
Male	All ages	191 (31.47)	125 (20.59)	323 (53.21)	320 (52.72)	93 (15.32)	162 (26.69)	< 0.0001***
(*N* = 607)	50–54	41 (35.65)	28 (24.35)	63 (54.78)	67 (58.26)	11 (9.57)	20 (17.39)	0.0002***
	55–59	47 (30.32)	26 (16.77)	85 (54.84)	88 (56.77)	23 (14.84)	41 (26.45)	< .0001***
	60–64	39 (29.77)	27 (20.61)	68 (51.91)	68 (51.91)	24 (18.32)	36 (27.48)	0.0001***
	65–69	33 (36.26)	26 (28.57)	45 (59.45)	40 (43.96)	13 (14.29)	25 (27.47)	0.0003***
	70 +	31 (26.96)	18 (15.65)	62 (53.91)	57 (49.57)	22 (19.13)	40 (34.78)	< 0.0001***
Female	All ages	38 (15.32)	16 (6.45)	103 (41.53)	60 (24.16)	107 (43.15)	172 (69.35)	< 0.001***
(*N* = 248)	50–54	16 (28.57)	7 (12.50)	28 (50.00)	20 (35.71)	12 (21.43)	29 (51.79)	< 0.001***
	55–59	9 (15.79)	3 (5.26)	32 (56.14)	20 (35.09)	16 (28.07)	34 (59.65)	< 0.001***
	60–64	7 (23.33)	4 (13.33)	13 (43.33)	8 (26.67)	10 (33.33)	18 (60.00)	0.0117*
	65–69	1 (2.63)	0 (0.00)	17 (44.74)	6 (15.79)	20 (52.63)	32 (84.21)	0.0033^b^**
	70 +	5 (7.46)	2 (2.99)	13 (19.40)	6 (8.96)	49 (73.13)	59 (88.06)	0.0046**

*Abbreviations*: *BMDRV* Bone mineral density reference value, *N* Number, *TDXA* T-score provided by the DXA manufacturer, *TNHA* T-score provided by a single reference population of the NHANES

^a^Chi-square test

^b^Fisher’s exact test

For *, **, and ***, the mean difference is significant at the 0.05, 0.01, and 0.001 levels, respectively

Statistical significance is set at *p* < 0.05

### Comparison of osteoporosis risk factors according to BMDRV

Table 5 presents multiple logistic regression analysis results to confirm the association with osteoporosis by deriving a significant risk factor for each category of osteoporosis prevalence according to the two BMDRV.

**Table 5:** Risk factor for each osteoporosis prevalence according to the two BMDRV

Variables	*N* = 821					
	Based on the TDXA			Based on the TNHA		
	OR	(95%CI)	*p*	OR	(95%CI)	*p*
Age	1.059	1.038–1.082	< 0.0001***	1.055	1.034–1.076	< 0.0001***
Sex						
Female	3.805	2.455–5.895	< 0.0001***	6.613	4.404–9.929	< 0.0001***
BMI	0.87	0.815–0.928	< 0.0001***	0.864	0.815–0.915	< 0.0001***
Smoking						
Former or Current	1.135	0.680–1.896	0.6275	1.187	0.772–1.824	0.4358
Alcohol						
Social or Binge	0.764	0.412–1.419	0.3944	0.664	0.397–1.110	0.1184
Time from injury						
Over 12 months	1.687	1.084–2.625	0.0205*	2.306	1.558–3.412	< 0.0001***
Etiology of injury						
Traumatic	0.689	0.447–1.061	0.0904	0.745	0.503–1.104	0.1425
Main diagnosis						
Tetraplegia	1.093	0.288–4.142	0.8964	1.609	0.450–5.746	0.4643
NLI			0.4487			0.3737
Cervical	0.454	0.110–1.863	0.2727	0.386	0.099–1.514	0.1723
Thoracic	0.764	0.436–1.339	0.3475	0.8	0.460–1.393	0.4308

*Abbreviations*: *BMDRV* Bone mineral density reference value, *BMI* Body mass index, *CI* Confidence interval, *NLI* Neurologic level of injury, *OR* Odds ratio, *TDXA* T-score provided by the DXA manufacturer, *TNHA* T-score provided by a single reference population of the NHANES

Reference groups are as follows: Male (Sex); Non-smoking (Smoking); Non-alcohol (Alcohol); <12 months (Time from injury); Non-traumatic (Etiology of injury); Paraplegia (Main diagnosis); and Lumbar (NLI)

For *, **, and ***, the mean difference is significant at the 0.05, 0.01, and 0.001 levels, respectively

Statistical significance is set at *p* < 0.05

As age increased by 1 year, the probability of osteoporosis prevalence increased by 5.9% (odds ratio [OR] = 1.059; 95% confidence interval [CI]: 1.038–1.082; *p* < 0.0001) and 5.5% (OR = 1.055; 95% CI: 1.034–1.076; *p* < 0.0001) of the TDXA and TNHA standards, respectively. Females had a higher probability of osteoporosis (or OZ ratio) than males, with the TDXA standards 3.805-times (95% CI: 2.455–5.895; *p* < 0.0001) and TNHA standards 6.613-times (95% CI: 4.404–9.929; *p* < 0.0001) higher than those of males. Moreover, the BMI increased by 1 kg/m^2^, and the probability of osteoporosis was 0.87 times the TDXA standard (95% CI: 0.815–0.928; *p* < 0.0001) and 0.864 times the TNHA standard (95% CI: 0.815–0.915; *p* < 0.0001). The probability of osteoporosis when the SCI period was > 12 months was also 1.687 times higher for the TDXA standard (95% CI: 1.084 − 2.625; *p* = 0.0205) and 2.306 times higher for the TNHA standard (95% CI: 1.558–3.412; *p* < 0.0001) than that when the SCI period was ≤ 12 months.

Therefore, regardless of the BMDRV criteria, the prevalence of osteoporosis increased with older age, female sex, lower BMI, and longer SCI period (*p* < 0.05). Additionally, the risk of osteoporosis was high for non-drinkers and individuals with non-traumatic injuries, tetraplegia, and lumbar injuries, without significant difference (*p* > 0.05).

## Discussion

We compared and analyzed osteoporosis diagnosis for patients with SCI aged ≥ 50 years according to region-based BMDRV of the DXA manufacturer and international BMDRV using the NHANES III data of females aged 20–29 years. The PONHA was significantly higher for males and females than the PODXA. Additionally, the lumbar spine and femoral neck were major osteoporosis diagnosis sites for the PODXA and PONHA, respectively. The risk factors for osteoporosis had different OZ ratios according to the two BMDRV criteria; however, the risk factors were similar based on older age, female sex, lower BMI, and longer SCI period, and no significant relationship was found between the different SCI-related clinical factors (*p* > 0.05).

The BMD of participants aged ≥ 50 years based on the diagnostic site of SCI was lowest in the femoral neck, and the same trend was observed for the T-score scale, regardless of the two BMDRV.

In the study of Lee et al. [28], the lumbar spine was identified as the highest diagnostic site for PODXA(Hological) in non-disabled Koreans aged ≥ 50 years, followed by the femoral neck and total hip. These findings are consistent with our study results. However, the femoral neck represented the highest diagnostic site for PONHA. SCI increases the risk of osteoporosis in the femoral neck due to biological, anatomical, and mechanical factors compared with that in the lumbar spine [22, 29–31]. This is because the spinal column is unaffected by demineralization (regardless of the time from injury) compared with the legs [30, 32, 33]. Biering-Sorensen and Schaadt [29] also reported that increased stress on the spinal cord from sitting in a wheelchair for a long time could have an osteogenic effect on the spine, thereby contributing to spinal BMD maintenance or increase. This explains the increase in the BMD of the lumbar area.

The consistency of the osteoporosis diagnosis according to the two BMDRV criteria was substantial for males and moderate for females. Additionally, a significant difference was found according to age and diagnosis site (*p* < 0.05), except for the prevalence of osteoporosis in the lumbar spine of males (*p* > 0.05).

Notably, the prevalence of osteoporosis differs according to the BMDRV [10–12]. For instance, the BMDRV of Caucasian females is high, consistent with the increased prevalence of osteoporosis in Caucasians compared to Asians [13, 14].

The significant risk factors for osteoporosis prevalence were similar, including older age, female sex, lower BMI, and longer SCI period (*p* < 0.05); however, the OZ ratio differed according to the BMDRV. Although not significant (*p* > 0.05), the risk of osteoporosis was high for non-drinkers and individuals with non-traumatic injuries, tetraplegia, and lumbar injuries.

The low bone mass (LBM) of individuals with SCI plays an important role in the vascular changes following the lesions of the automatic nervous system rather than immobilization [18, 22, 34]; the NLI determines the extent of damage caused by calcium desorption (demineralization) rather than the intensity of osteoporosis [23, 30, 32–36]. Additionally, veins and capillaries stagnate due to the desorption of the sympathetic nervous system, thereby reducing the gas exchange and bone nutritional supply and transforming mesenchymal cells into osteoblasts cells [34, 35].

Therefore, the main LBM factor in SCI was the physiopathology of the condition, and the correlation with SCI-related clinical factors showed mixed results, except for the SCI period.

Patients with SCI have a higher risk of osteoporosis than those without disabilities due to pathophysiological causes, particularly patients with older age, female sex, lower BMI, and a longer SCI period. Additionally, when osteoporosis was diagnosed using the TNHA standard, a tendency similar to that of the major LBM reduction site in SCI was observed. Therefore, osteoporosis diagnosis in individuals with SCI should be considered in addition to the regional base, and related studies are required. Overall, patients with SCI are at a high risk of osteoporosis compared to those without disabilities. If strict standards are considered, employing the international BMDRV is necessary for diagnosing osteoporosis in individuals with SCI in Korea rather than the regional-based BMDRV.

This study had some limitations. First, although the Lunar BMD values were converted to the Hologic BMD values for the TDXA derivation, the T-scores derived from each manufacturer were used in their original form without conversion. Second, osteoporosis diagnosis in individuals with SCI should not be based on BMD alone; therefore, other factors, such as complications, lifestyle, range of activity, and drug use, should be considered depending on the SCI characteristics [9]. However, these complex variables were not considered in this study, and further studies are needed. Third, the official position statement of the International Society for Clinical Densitometry recommends that DXA tests should be performed for the total hip, proximal tibia, and distal femur of patients with SCI [7]. However, this study focused on the diagnostic standards for osteoporosis used in clinical practice, including the general public, and BMD measurements were performed at the lumbar spine, femoral neck, and total hip, as recommended by the official position statement of the International Society for Clinical Densitometry for adults [6]. Therefore, further studies focusing on the proximal tibia and distal femur, which have a high risk of osteoporosis fracture in individuals with SCI, should be conducted.

## Conclusions

The diagnostic site and prevalence of osteoporosis in individuals with SCI differed according to the regional-based TDXA and international standards of the TNHA. Therefore, to prevent osteoporosis in individuals with SCI, further studies on the BMDRV are needed to establish accurate diagnostic criteria. This is the first study to identify the prevalence, diagnostic site, and risk factors of osteoporosis in Korean adults with SCI aged ≥ 50 years by comparing BMDRV, thereby reflecting their significance.

## Data Availability

The data that support the findings of this study are available from EMR (Electronic Medical Record) and PACS (Picture Archiving and Communication System) in the National Rehabilitation Center, but restrictions apply to the availability of these data, which were used under license for the current study, and so are not publicly available. However, data are available from the author (Ohyoo Kim) upon reasonable request and with permission of EMR (Electronic Medical Record) and PACS (Picture Archiving and Communication System) in the National Rehabilitation Center.

## Abbreviations

AIS: American Spinal Injury Association impairment scale
BMI: Body mass index
BMD: Bone mineral density
BMDRV: Bone mineral density reference values
DXA: Dual-energy x-ray absorptiometry
NHANES: National Health and Nutrition Examination Survey
NLI: Neurologic level of injury
NRC: National Rehabilitation Center
PODXA: Prevalence of osteoporosis according to the T-score provided by the DXA manufacturer
PONHA: Prevalence of osteoporosis according to the T-score provided by a single population of the National Health and Nutrition Examination Survey
SCI: Spinal cord injury
SD: Standard deviation
TDXA: T-scores provided by DXA manufacturer
TNHA: T-scores provided by the NHANES III
WHO: World Health Organization
